# Salutogenic Affordances and Sustainability: Multiple Benefits With Edible Forest Gardens in Urban Green Spaces

**DOI:** 10.3389/fpsyg.2018.02344

**Published:** 2018-12-04

**Authors:** Jonathan Stoltz, Christina Schaffer

**Affiliations:** Department of Physical Geography, Stockholm University, Stockholm, Sweden

**Keywords:** salutogenic affordances, multiple-use, urban green spaces, green densification, sustainability, agroforestry, edible forest gardens

## Abstract

With increased urbanization, ecological challenges such as climate change and loss of biodiversity, and stress-related disorders globally posing a major threat to public health and wellbeing, the development of efficient multiple-use strategies for urban green spaces and infrastructures is of great importance. In addition to benefits such as climate and water regulation, food production, and biodiversity conservation, green spaces and features have been associated with various health and wellbeing outcomes from a psychological perspective. Research suggests links between exposure to green environmental qualities and restoration from psycho-physiological stress and attention fatigue, promotion of physical activity, increased neighborhood satisfaction and even reduced mortality. Especially strong associations have been observed in urban and socio-economically challenged areas. Usually such salutogenic, i.e., health-promoting, effects are explained through theories related to the notion of *biophilia*, i.e., the idea that humans share innate tendencies to attend to natural environments and features that have been beneficial during evolution. This paper assumes an ecological approach to perception and behavior to be fruitful in order to analyze the salutogenic potential of environments such as urban green spaces and to step beyond the “green vs. gray” dichotomy that has been prevalent through much of the research on health-promoting environments. Through an analysis of environmental affordances for certain perceived qualities such an approach is explored through a proposed concept for urban green space use and management, *the edible forest garden*. Such gardens, based on agroecological principles, have emerged as one of the most promising models regarding ecologically sustainable food production. In addition to potential contributions of importance for urban sustainability and biodiversity, we argue that the inclusion of edible forest gardens in urban green spaces – today globally dominated by lawns – also potentially could reinforce several affordances of salutogenic importance, both in terms of, e.g., social cohesion but also in regard to restoration from psycho-physiological stress and attention fatigue. Increased opportunities for contact with nature and processes of food production may also reinforce pro-environmental behaviors in the population and thus also affect long-term sustainability.

## Introduction

More than half of the human population resides in urban settings, and urbanization is an ongoing trend (WHO, 2014). By 2050 66% of the world’s population is expected to be urban, as compared to 30% a hundred years before, in 1950 (ibid). Urbanization thus poses a major current and future challenge that affect how people interact with their close living environment, including potentially diminished contact with the natural world in terms of both quality and quantity (Markevych et al., 2017). Robert Pyle remarked that “local and tacit knowledge related to agriculture is disappearing from metropolitan landscapes, creating an ‘extinction of experience’ of human–nature interaction and a collective ‘forgetting’ of how to grow food” (Pyle, 1978). Such an experiential lack may lead to a degradation of public health and wellbeing, a loss of emotional affinity to nature and a decline in pro-environmental attitudes (Soga and Gaston, 2016). It has also been shown that various mental disorders, such as depression and even schizophrenia, are more common in urban than in rural areas (Peen et al., 2010). This has been attributed to higher stress levels in urban settings, and brain imaging studies have suggested that residents of urban areas often have a lesser capacity to cope with stress than rural dwellers (Lederbogen et al., 2011).

Meanwhile, non-communicable diseases such as cardiovascular disease, stroke, diabetes type 2, obesity, stress-related mental disorders, depression, and anxiety dominate the global disease burden and both insufficient physical activity and chronic stress are recognized as risk factors for such disorders (WHO, 2010). In Sweden the trend of sick leave due to mental health problems is increasing and according to a Swedish Social Insurance Agency (2013) report, the most common cause of sickness absence from work was stress-related mental illness. Globally, mental health problems are estimated to be among the major contributors to ill health and work disabilities (Salomon et al., 2012; Vos et al., 2013). A lack of green space access in urban areas have been linked to more self-reported mental distress and greater rates of anxiety and depression (Maas et al., 2009; van den Berg et al., 2010; Nutsford et al., 2013), as well as premature death (van den Bosch and Bird, 2018). The latter link applies to all-cause mortality but in particular to increased mortality in cardiovascular diseases (van den Berg et al., 2015; Egorov et al., 2016). Such findings could partly be explained by reduced green space access leading to decreased opportunities for physical activity (e.g., Konijnendijk et al., 2013) and restoration from high stress levels (Hartig et al., 2014; Braubach et al., 2017). Especially pronounced effects have been observed for people with lower incomes (Mitchell and Popham, 2008), highlighting the potential of using urban green spaces as a means to mitigate health inequalities in socioeconomically challenged areas, as discussed by e.g., Skärbäck et al. (2014). In addition, Hanski et al. (2012) have suggested that the reduced biodiversity in urban settings also may lead to decreased diversity of gut and skin microbiota. This in turn has been associated with inflammatory conditions, including asthma, allergic and inflammatory bowel diseases (IBD), type1 diabetes, and obesity (Haahtela et al., 2013).

## Potential Benefits of Urban Green Spaces

Urban green spaces and infrastructures may contribute to the reduction of noise, filtering of air, and to the adaptation of climate change effects such as regulation of temperature, water run off, function as carbon sinks, while simultaneously serve various aesthetic and social purposes (e.g., Bolund and Hunhammar, 1999; Berghöfer et al., 2011; Gómez-Baggethun et al., 2013). Such functions is part of the Nature Based Solutions approach suggested by the European Commissions, and is often less expensive than technical solutions (Bauduceau et al., 2015). Regarding adaption benefits it seems that a heterogeneous vegetation structure is preferable and that trees with large and dense canopies are the most effective for both cooling and rainfall interception (see Brink et al., 2016). In addition to climate change, recent research also reveals an exceptionally rapid decline of plant and animal populations over the last century due to human actions (Millennium Ecosystem Assessment, 2005; Ceballos et al., 2015, 2017). Habitat loss is considered a main driver of this development, and although urbanization has had a negative impact on many species urban areas can support native biodiversity and even threatened species (Hall et al., 2016; Ives et al., 2016). Due to the possibilities of rapid development and change, urban green spaces may provide opportunities for instant and continuous creation of new habitats (Beninde et al., 2015).

**FIGURE 1:**
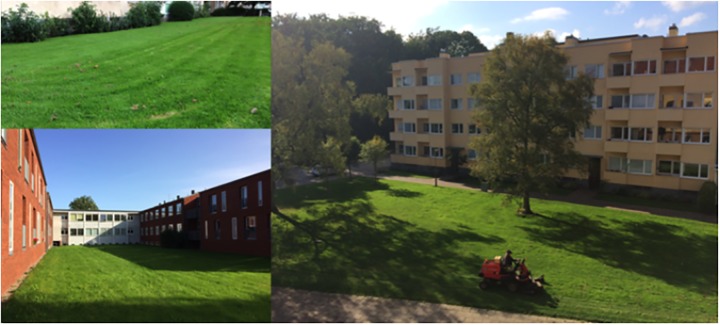
Lawns dominate urban green spaces globally.

Meanwhile, lawns dominate urban green spaces (Figure 1) and occupy around 70–75% of such areas globally (Ignatieva, 2010; Ignatieva et al., 2015). In Sweden, close to 25% of the cities are covered by lawns according to Hedblom et al. (2017) and it has been suggested that lawns contribute to increasingly uniform urban environments around the world (Ignatieva, 2010). In addition, traditional lawns are expensive and resource demanding to manage and rather poor in terms of biodiversity (ibid). They are green, but may in spite of this be rather weak regarding support for some important human needs, such as restoration from attention fatigue and psycho-physiological stress. There is a need for development of strategies that allow for urban environments and green spaces to be efficiently used in order to simultaneously meet the current ecological and social challenges. The development and employment of such multiple-use strategies for urban green spaces and infrastructures may be seen as a process of “green densification”, aimed to optimize the design and planning of such features in order to provide multiple benefits addressing the various current challenges.

## Moving Beyond the “Green Vs. Gray” Dichotomy With a Salutogenic Perspective

The term “salutogenesis” describes an approach focusing on factors that support human health and wellbeing, rather than on “pathogenesis,” i.e., factors that cause disease (Antonovsky, 1996). The relationship between health and disease is seen as a continuum rather than as a dichotomy. Individual or environmental factors that push an individual toward the disease end of this continuum are termed *stressors* and factors that work in the opposite direction, toward optimal health and wellbeing, are called *salutogens*. According to Antonovsky, human health and wellbeing ultimately depend on the individual’s ability to create and maintain a “sense of coherence” and meaning, thus strengthening the capacity to cope with life’s various stressors (ibid). Salutogenic strategies, aimed at supporting such processes, may then complement pathogenic strategies that primarily strive to mitigate or eliminate stressors (Antonovsky, 1996; Becker et al., 2010).

Markevych et al. (2017) suggest that beneficial effects on human health and wellbeing from natural environments and green spaces work through three main complementary pathways; (1) *mitigation* (“reduction of harm,” e.g., reducing exposure to air pollution, noise and heat, etc.), (2) *restoration* (“restoring capacities,” e.g., attention restoration, physiological stress recovery, etc.), and (3) *instoration* (“building capacities,” e.g., encouraging physical activity, facilitating social cohesion, etc.). In the light of salutogenic theory, mitigating strategies could be considered as primarily pathogenic, i.e., focused on harm-reduction, whereas support of restorative and instorative pathways could be considered as fundamentally salutogenic, i.e., focused on restoring and strengthening the capacities needed to cope with life’s various stressors and ultimately facilitating a sense of coherence and meaning in life. Salutogenic pathways could arguably be seen as distinguished in comparison to most mitigating services in that they primarily depend on environmental support for certain experiences and behaviors, i.e., rely on a level of analysis that takes human psycho-physiological needs and preferences into account in order to be properly understood.

Although existing reviews and meta-analyses (e.g., Egorov et al., 2016) seem to confirm causal relations at population level between various beneficial health outcomes and access to natural environments and green spaces, Markevych et al. (2017) also highlight the fact that some epidemiological studies have failed to support such connections. Such findings might indicate that green spaces can support salutogenic pathways to different degrees. Furthermore, salutogenesis can also include more subtle effects of, e.g., aesthetic appreciation that may or may not be visible in epidemiological studies, arguably often focused on less subtle health and wellbeing outcomes. The same is arguably also true for various urban/built environments and psychologically relevant qualitative differences may exist here as well, as highlighted by, e.g., Stigsdotter et al. (2017a). Sallis et al. (2016) report that, in addition to the number of parks, the population density, intersection density, and public transport density all were positively related to physical activity in urban contexts in several cities across multiple countries and continents. It thus seems clear that both “green” and “gray” environments and features may function as salutogens in various ways and that research in health-promoting environments need to move beyond this dichotomy, arguably until recently prevalent in the field. There is thus a need to identify in more detail the specific qualities important in order for different environments to support salutogenic processes efficiently. This paper focuses specifically on urban green spaces and qualities within these that may contribute to their potential as salutogens in people’s lives. This is done without thereby dismissing the importance of other urban qualities.

## Affordance Theory to Analyze the Salutogenic Potential of Urban Green Spaces

We believe that much of the salutogenic potential of environments could be understood through an ecological approach to perception and behavior, by analyzing the environmental support for certain *affordances* in people’s living environment. Introduced by Gibson (1979), an affordance is regarded as a perceivable and utilizable possibility for a certain behavior or experience, provided to individuals by environments. We here consider affordances primarily as relations between the individual and the environment, in accordance with the affordance theory developed by Chemero (2003, 2009). As such they are situation-dependent and are shaped between the abilities and needs of the individual and the present socio-material environmental conditions. Previous studies have applied the affordance concept to investigate how outdoor environments can afford, e.g., physical activity levels (e.g., Cosco, 2006; Björk et al., 2008) and independent mobility (Kyttä, 2003), socialization (Clark and Uzzell, 2002), self-regulation, (Korpela et al., 2002) and play behaviors (e.g., Heft, 1988; Zamani and Moore, 2013) among children. Kyttä (2002) revealed rural environments to have higher potential in providing affordances for play and social behaviors among children than urban environments. Other studies have used an affordance approach to investigate the potential of different environmental settings to aid in the restoration of stress and stress-related illness, such as rehabilitation gardens (e.g., Stigsdotter et al., 2017b) and forest environments (Stoltz et al., 2016).

Understood as dynamic human-environment relations, the affordances perceived are affected by various aspects regarding individual needs and characteristics, social factors, and physical environmental conditions. For instance, the perceived neighborhood safety may be regarded as one important factor that may shape the perception and utilization of green space affordances, as shown through epidemiological research by, e.g., Weimann et al. (2017). Such results indicate the importance of accounting for the broader socio-material context to understand how green space affordances are shaped and utilized. In general, salutogenic effects from urban green spaces have been related primarily to the amount of time spent there (Grahn and Stigsdotter, 2003) and research has shown both the use rate and time spent in urban green spaces to decrease markedly already in the interval of 100–300 m away from the dwelling (Grahn and Stigsdotter, 2003; Nielsen and Hansen, 2006). It is thus important to identify factors that make urban green spaces afford actual use and it seems clear that accessibility, not the least through physical proximity, is key in this regard. The perceived biodiversity of urban green spaces has been identified as another important factor for visit rates (Gyllin and Grahn, 2015; Sandifer et al., 2015; World Health Organization [WHO], 2015), thus also indicating a general importance for the qualities perceived within green spaces. In order to analyze the salutogenic potential of urban green spaces in more detail, however, and possibly come up with evidence-based design and management suggestions, we would need a deeper understanding for the qualities of such environments that are important in shaping affordances of salutogenic significance. Also from a planning perspective this would be important to be able to identify which needs that are well catered for in a given environment and those that might require improved environmental support.

## Natural Environments as a Salutogenic Factor

Human connections and interactions with green and natural environments have been the focus of much research from various perspectives. Physical, mental, and spiritual perspectives have been highlighted and associated with various health and wellbeing outcomes. Research has described how perceptions of natural environments and features may impact various aspects of human health and wellbeing (e.g., Ulrich, 1984; Nilsson et al., 2011; Haluza et al., 2014), cognitive functions (e.g., Kaplan, 1995; Ottosson and Grahn, 2005; Berman et al., 2008) and stress-related aspects such as parasympathetic nervous system activity (Annerstedt et al., 2013), cortisol levels (Ward Thompson et al., 2012; Roe et al., 2013) and blood pressure and heart rate (e.g., Ottosson and Grahn, 2005). Such environmental influence has also been studied in various kinds of rehabilitation contexts (e.g., Ottosson, 2001; Pálsdóttir, 2014) and nature-based rehabilitation for individuals with stress-related disorders has been performed in various settings (e.g., Sonntag-Öström et al., 2014, 2015; Pálsdóttir et al., 2017; Stigsdotter et al., 2017b). Sahlin (2014) describes how such environments could promote and facilitate high-order cognitive behaviors such as existential reflections that aid in shaping experiences of meaning, coherence, and acceptance. Influence of natural and green environments has also been studied from a children’s perspective. Mårtensson et al. (2013) for instance investigated relations to physical activity among school children. Carrus et al. (2015) showed how contact with nature could positively influence both cognitive capacities and social behavior among preschool children.

### The Psycho-Evolutionary Theory; Restoration From Stress

Commonly, such effects from natural environments and features are explained with theories related to the *Biophilia*-hypothesis (Wilson, 1984; Ulrich, 1993), i.e., the idea that humans tend to respond in favor to natural characteristics that have been beneficial to survival and wellbeing during human evolution. The often-cited *psycho-evolutionary* theory (PET; Ulrich, 1986; Ulrich et al., 1991) focuses mainly on restoration from psycho-physiological stress. It holds that immediate affective responses, to a large degree dependent on common evolutionary traits, are important for how we respond to different environments. Responses of approach or avoidance depend on how environmental perceptions are interpreted and valuated in regard to survival and wellbeing, much in line with the evolutionary approach to motivation and valuation suggested by Mercado-Doménech et al. (2017). In accordance with Orians (1986); Ulrich (1986) suggests that our genetic configuration explains a preference for “savannah-like” environments consisting of layered vegetation with a mix of trees, grasses, and shrubs, preferably with visible water features, as well as support for the “prospect/refuge” dimension, i.e., opportunities for sheltered overviews and outlooks, as previously proposed by Appleton (1975). Empirical evidence in support of these theoretical claims has been reported by, e.g., Falk and Balling (2010). Such environmental characteristics are suggested to trigger stress-reducing responses whereas threatening or adverse conditions may induce stress (Ulrich et al., 1991). In general, PET suggests urban environments and stimuli to be significantly more stressful and less restorative than natural settings and features (ibid).

### The Attention Restoration Theory; Restoration From Attention Fatigue

Another influential model in the field is the *attention restoration* theory (ART; Kaplan and Kaplan, 1989; Kaplan, 1995). It shares with PET the basic idea that evolutionary traits play an important role in how humans perceive and react to environments. Instead of psycho-physiological stress, however, ART instead focuses on our capacities for attention where it distinguishes between two basic kinds; “directed attention” and “soft fascination.” ART suggests that our directed attention has a limited capacity and gets exhausted if overused. Typically the use of executive functions, such as planning and problem solving, require the activation of directed attention (Kaplan and Berman, 2010), as do many urban environments with an abundance of signals, information, and noise that the brain needs to sort through and handle. Circumstances that instead trigger our soft fascination, or “spontaneous” attention, e.g., certain natural environments and features according to ART, allow our directed attention to rest and its capacities to restore (Kaplan, 1995). In order for such restoration to occur, ART suggest that the environment should: (1) offer a sense of *being away* from the everyday environment, (2) give a sense of *extent*, of an uninterrupted world in itself, (3) offer opportunities for *fascination*, through, e.g., natural features, and (4) be *compatible* with individual needs and abilities (Kaplan and Kaplan, 1989). ART, however, does not go into further detail in explaining how environments need to be physically structured in order to support these factors at the level of planning and design of public environments and urban green spaces.

## The Supportive Environment Theory; an Ecological Approach

The *supportive environment theory* (*SET*; Grahn et al., 2010) represents an approach to account for restorative and instorative processes (Stigsdotter and Grahn, 2003) that acknowledges the basic mechanisms and pathways suggested by both PET and ART, but emphasizes human’s embodied relations with the environment and its affordances for certain experiential qualities termed *perceived sensory dimensions*. The theory suggests eight such qualities to be of particular importance to account for salutogenic effects. These have been revealed through factorial analysis of several different survey studies regarding people’s green space preferences and use. They are based on people’s reported needs regarding environmental support in their daily lives and do not rely on, e.g., image studies which has otherwise been common in the field. They may thus be regarded as ecologically valid categories in terms of green space qualities of potential salutogenic importance. Grahn and Stigsdotter (2010) term these qualities as (1) *Serene*, (2) *Nature*, (3) *Rich in species*, (4) *Space*, (5) *Prospect*, (6) *Refuge*, (7) *Culture*, and (8) *Social*. Table 1 presents brief descriptions of each perceived sensory dimension.

**Table 1:** Eight perceived sensory dimensions associated with affordances supporting different needs.

Perceived sensory dimension	The environment affords behaviors/experiences associated with…
(1) Serene	Peace, silence and care. Sounds of nature. Freedom from disturbances.
(2) Nature	Fascination with the natural world; the “self-made” as opposed to the man-made. Seemingly self-sown plants, a sense of untouched nature.
(3) Rich in species	A sense of abundance and variation, a large diversity of different species of plants and animals.
(4) Space	An experience of entering a world in itself, a coherent whole.
(5) Prospect	Views of the landscape, a sense of openness, prospects, vistas and stays.
(6) Refuge	Shelter and safety. Possibilities to relax and, e.g., let children play freely.
(7) Culture	A sense of fascination with human culture and history, the course of time and human efforts.
(8) Social	Social activities and interactions.


*After Grahn and Stigsdotter (2010).*

Each dimension indicates a generally perceived need that requires support in the environment (Grahn and Stigsdotter, 2010; Grahn et al., 2010) and people tend to agree as to which level an environment support a quality or not, making them suitable for objective environmental evaluations (see e.g., de Jong et al., 2011, 2012; Stoltz et al., 2016). Such general agreement may be important in the context of design and planning of public environments such as urban green spaces where individual tailoring is not applicable. It may also be considered as in line with the notion that humans share certain tendencies regarding environmental preferences due to common evolutionary traits, as held by PET and ART. A key assertion of SET, however, is that preferences and valuations (Mercado-Doménech et al., 2017) of each quality vary with changing needs, depending on, e.g., stress levels (Grahn et al., 2010). This has also been clear when studied in various rehabilitation contexts. For instance, Pálsdóttir et al. (2017) investigated which qualities that were considered the most restorative in a rehabilitation forest environment. The results showed the perceived sensory dimensions Serene, Space, Refuge, and Nature to be rated highest in this regard and the Social quality to generally be seen as the least restorative, all in line with previous studies (e.g., Grahn and Stigsdotter, 2010). This indicates that salutogenic design and planning of urban green spaces should take into account the need for variation in terms of perceived environmental qualities in order to satisfy different needs in the population.

The perceived sensory dimensions may thus be considered as quite stable in the environment regarding their general presence/support, while their actualization as perceived affordances will vary depending on individual needs. This makes them interesting as a framework through which affordances of salutogenic importance, although always realized as unique human-environment relations, may be considered in a more general and objective sense for purposes of design, planning and evaluation of public environments. Following these assertions, public health and wellbeing outcomes may to some degree depend on the affordances for the different perceived sensory dimensions in people’s close living environment. Such relations have been investigated in epidemiological studies. Björk et al. (2008) found an association with the number of dimensions perceived as supported in the neighborhood green spaces and reported neighborhood satisfaction. The opposite association was found regarding body mass index (BMI; ibid). These effects were, perhaps not surprisingly, most pronounced among tenants as compared to house-owners. de Jong et al. (2012) found an association with increased physical activity and the number of supported dimensions in the neighborhood green spaces. These results were all adjusted for in regard to individual characteristics such as age, sex, educational level, and income, suggesting that the observed effects indeed share a common driver in the structure of the physical neighborhood environment. In line with such findings, Stigsdotter et al. (2017b) suggest that the perceived sensory dimensions framework is valid for use as a guideline in the design and evaluation of salutogenic environments.

## The Edible Forest Garden

**FIGURE 2:**
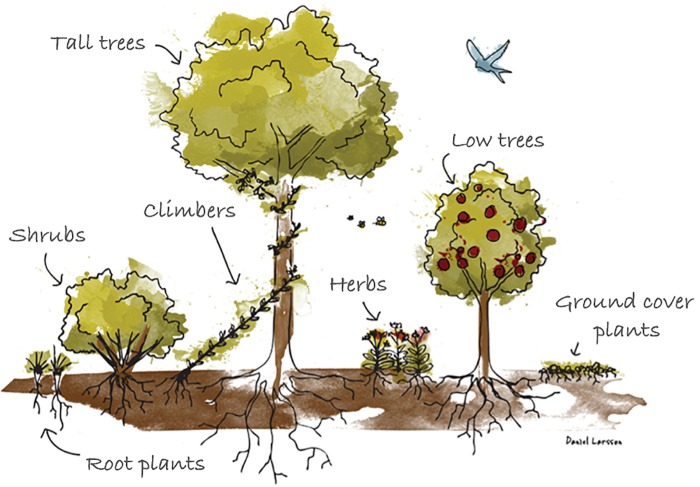
The multi-strata (layered) system of an edible forest garden (illustration by Daniel Larsson).

*The edible forest* is one of several agroforestry practices based on *agroecological* principles (Gliessman, 2007). Agroecology is a scientific discipline derived from agronomy and ecology that studies productive lands through an understanding of the workings of natural ecosystems (ibid). The edible forest garden describes a low maintenance, productive and species rich cultivation system with its origins in the tropics (Hart, 1996; Crawford, 2010). It is modeled after the structure of young natural woodland and consists of edible perennials such as fruit and nut trees, shrubs with berries, herbs, vegetables, flowers and fungi that are intercropped in layers in a so-called *multi strata* system (Figure 2). The edible forest garden thus resembles a forest more than a conventional horizontal garden and the management methods used mimics the cycles in natural ecosystems (Crawford, 2010). No external inputs of resources such as irrigation, pesticides, or fertilizers are used and digging/tilling techniques are avoided. Instead recycling of organic matters on the ground makes the soil fertility self-generative, the moist is kept and green house gas emissions are low or even negative (ibid). Natural pest control is accomplished through the high species richness – usually about 100–200 species per garden – and the forest garden is also resilient of weather extremes as well as demanding lesser labor for maintenance and weeding than annual crops (ibid). Edible forest gardens exist as home gardens in the tropics (Bardhan et al., 2012) and in temperate areas such as in the United Kingdom since a few decades (Hart, 1996; Crawford, 2010). In Sweden, their ecological benefits have been highlighted through an applied pilot project on 13 smallholdings (Björklund et al., 2018).

### Edible Forest Gardens and Urban Sustainability

From the literature on food production the edible forest garden is considered as promising regarding ecological sustainability (Crawford, 2010). Russo et al. (2017) include edible forest gardens in their concept of “edible green infrastructures” and the city of Seattle, United States, has an ambitious tree-planting program in order to create edible urban landscapes supporting urban sustainability (McLain et al., 2012). Clark and Nicholas (2013) have investigated 37 existing urban “fruit forests” in the United States and address their multiple benefits regarding sustainability. Furthermore, edible forest gardens can increase urban biodiversity; even a small bed could consist of +100 species and this high biodiversity could contribute to ecological values, especially when compared to traditional lawns (cf. e.g., Ignatieva, 2010; Ignatieva et al., 2015). Since it consists of trees and shrubs the overall structure resembles a forest/orchard with shelters, providing habitats for organisms such as birds and insects (Björklund et al., 2018). Even smaller forest gardens (≈60 m^2^) can exhibit these qualities (ibid) and thus contribute to increased urban biodiversity. On the landscape level edible forest gardens could strengthen the green infrastructure through contributing to ecological connectivity (Russo et al., 2017). Bohn and Viljoen (2011), under the concept of the Continuous Productive Urban Landscape (CPUL), have suggested cities to have continuous productive stretches with room for green areas, mobility without vehicles, and urban agriculture. Edible forest gardens could be part of such a strategy.

**FIGURE 3:**
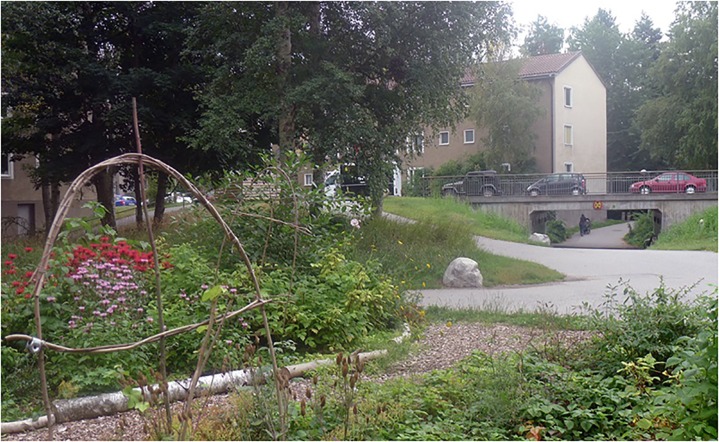
View from a young edible forest garden in Bagarmossen, south of Stockholm, Sweden. It is located close to the metro station and a shopping mall in an area with apartment blocks without own gardens.

To our knowledge there are not yet any published literature on temperate zone urban edible forest gardens. We suggest, however, that they have great potential in these areas as well. They are more robust than annual cropped gardens and therefor allow other activities such as room for play or for people that do not want to garden themselves. Edible forest gardens could be integrated in the ordinary maintenance of outdoor-areas performed by public (e.g., municipalities) or private (e.g., housing companies) actors. They demand less labor, resources, and land area than annual cropped gardens (Hemenway, 2009; Crawford, 2010; Björklund et al., 2018) and could therefor also be less expensive. Edible forest garden could thus be an alternative for municipalities with constrained budgets. Stockholm for instance is a segregated city (Bremberg et al., 2015) and since the municipality owns 70% of the land forest gardens could contribute to urban sustainability in underprivileged districts by urban agriculture in the forms of edible forest gardens and community gardens. In a small-scale study on edible forest gardens in residential areas in Stockholm, Schaffer (2016) highlighted the multiple user-groups that visited the gardens, thus indicating a potential for broad social benefits. If space is limited, forest gardens can be kept small and fit in well in existing urban environments, e.g., in between apartment blocks (Figure 3).

## Community Gardening in Urban Areas

Social factors such as neighborhood interaction patterns, social cohesion (Wilkinson, 1996), social capital (Giordano et al., 2012), and a shared sense of coherence and safety (Taylor et al., 1997) have important influences on health and wellbeing. Urban agriculture in general is suggested to contribute to multiple dimensions of sustainability depending on organizational form, location, size, and gardening methods (Guitart et al., 2012; Mok et al., 2014; Eigenbrod and Gruda, 2015). As an organizational form the *community garden* have been highlighted in the literature. Hale et al. (2011) argues that the relational qualities from community gardening contributes to social health, since it nurtures relations such as those between gardener-plant, gardener-gardener and the garden/gardener-the local community. Community gardening could increase the social capital (Firth et al., 2011) and contribute to learning and a sense of place (Bendt et al., 2013). Gardening as an activity also contributes to health for gardeners from a physical activity perspective, and for others as well since the garden could be the attraction for a trip or a walk (Hale et al., 2011). Various forms of urban gardens, such as edible forest gardens, allotment gardens, etc., organized as community gardens may contribute to social capital (Firth et al., 2011) and a sense of place (Bendt et al., 2013). In a recent study by Bonow and Normark (2018) on community gardens in Stockholm, the many social qualities generated are highlighted versus the rather small amount of food produced.

Clavin (2011) identified features of sustainable design in community gardens in the United Kingdom that had impact on wellbeing. Factors that affected wellbeing were related to agency (both individual and collectively) from experiential learning (learning by doing) and having choice (freedom) to work in one’s own manner (to be both slow or busy, to be both alone or work with others), and having choice of a variety of tasks suitable for different people different days. Community gardens as a form of do-it-your-self urbanism (Finn, 2014) enable people to participate in the design of their own neighborhood. Such gardens might thus afford an arena where urban citizens can be more than voters/consumers, but also actively engage as co-creators of the city. In allotment areas ecological knowledge is shared among gardeners and over generations (Barthel et al., 2010) and this could also be true for community-based forest gardens. Edible forest garden may also afford learning opportunities, as described by, e.g., Askerlund and Almers (2016) who studied how edible gardens could support children’s learning on ecology. When located in urban areas edible forest gardens may provide increased possibilities of interaction with the natural world, thus aiding an increased sense of connectedness to nature (Hale et al., 2011), support environmental awareness and pro-environmental behaviors (cf. e.g., Annerstedt van den Bosch and Depledge, 2015). Arguably, forest gardens could thus somewhat remedy the “extinction of experience” mentioned by Pyle (1978).

## Perceived Sensory Dimensions of Edible Forest Gardens

We argue that edible forest gardens in urban green spaces is an interesting concept to explore, both in regard to such dimensions of ecological and social sustainability as has been outlined above, and in terms of affording perceived qualities of salutogenic importance, which could be highlighted using the perceived sensory dimensions framework described above. Compared with lawns, edible forest gardens seems particularly promising in supporting perceived sensory dimensions such as Nature, Rich in species, and Refuge; dimensions that have been described in the literature as important to support restorative processes (e.g., Grahn et al., 2010; Pálsdóttir et al., 2017; Stigsdotter et al., 2017b). Table 2 relates typical features of the edible forest garden with each perceived sensory dimension of the SET theory.

**Table 2:** Typical features of edible forest gardens in relation to eight perceived sensory dimensions (after Grahn and Stigsdotter, 2010).

Perceived sensory dimension	The environment affords behaviors and experiences associated with…	In relation to features of the typical edible forest garden
(1) Serene	Peace, silence and care. Sounds of nature. No disturbances.	Edible forest gardens, especially when mature, could provide habitats that attract singing birds and humming insects. Sounds of wind blowing through the trees etc. could also reinforce affordances associated with this dimension.
(2) Nature	Fascination with the natural world; the “self-made” as opposed to the man-made. Plants seem self-sown, a sense of untouched nature.	Mimicking the natural ecosystems of young woodlands, the mature edible forest garden could provide plenty of affordances associated with this dimension, e.g., trees and plants with interesting shapes, a sense of nature’s power to grow and create through the passing of time.
(3) Rich in species	A sense of abundance and variation. A large diversity of different species of plants and animals.	Edible forest gardens typically exhibit a very high biodiversity. Usually +100 plant species, most of them edibles. The forest garden environment could also attract various animals through the different habitats created by the various plants and the young woodland, *multi-strata* structure.
(4) Space	An experience of entering a world in itself, a coherent whole.	May be reinforced through the *multi-strata* structure the forest garden, adding to a sense of 3-dimensional “spaciousness” and of entering into “another world, a coherent whole.” An entrance gate may further strengthen such affordances. It would, however, be important for the forest garden to be large enough in order to fully support associated experiences and behaviors (e.g., “wandering around”).
(5) Prospect	Views of the landscape, a sense of openness, prospects, vistas and stays.	Affordances associated with this dimension are generally better reinforced by, e.g., lawns rather than by edible forest gardens in themselves. However, from a distance the forest garden might provide for a pleasant “view” or “scenery” that are important aspects of this dimension.
(6) Refuge	Shelter and safety. Possibilities to relax and, e.g., let children play freely.	Could be reinforced through the *multi-strata* structure of the forest garden with trees and shrubs of various heights mixed with more open parts. A gate to the garden may further strengthen affordances that allow for a sense of shelter and privacy and to “see without being seen.”
(7) Culture	A sense of fascination with human culture and history, the course of time and human efforts.	An edible forest garden represents a highly cultivated environment. Crops could be chosen that relate to cultural heritage. With time a growing sense of appreciation for the history of the place and the human labor put into the garden might grow, thus further strengthen associated affordances.
(8) Social	Social activities and interactions.	Especially true when realized as community gardens in public green spaces close to dwellings. Opportunities for learning, workshops, gardening activities, etc. have been highlighted in the literature.


The use of trees and other perennials, a core principle of agroforestry, could be an efficient means to reinforce affordances for the perceived sensory dimension of Nature; especially so when given an impression of being “self-sown” (Grahn and Stigsdotter, 2010). The general salutogenic potential of urban trees in particular have been highlighted in previous research (e.g., Kardan et al., 2015), as have the salutogenic potential of forest environments (e.g., Sonntag-Öström et al., 2014, 2015). In addition, trees can often be made visible from the windows of houses that in dense urban areas often reach several floors above ground and thus increase the need for vertical green structures in order to be visible from inside the dwellings. The salutogenic potential of having access to trees outside the window has not the least been highlighted by Ulrich (1984) in a well-known study. Support for the perceived sensory dimension of Nature would possibly increase over time as the forest garden grows and matures; a sense of nature’s “untouched” development over time is indicated as important to strengthen this perceived quality (Grahn and Stigsdotter, 2010). Compared to, e.g., lawns – that arguably will look almost the same even after 50 years of cultivation – a forest garden with 50-year-old fruit and nut trees would give quite another impression in such terms. Experiences of the passage of time in nature could further be reinforced through the high biodiversity of the forest garden with a large variety of plants that may mature at different times during the season, thus changing the environmental impressions as time passes.

**FIGURE 4:**
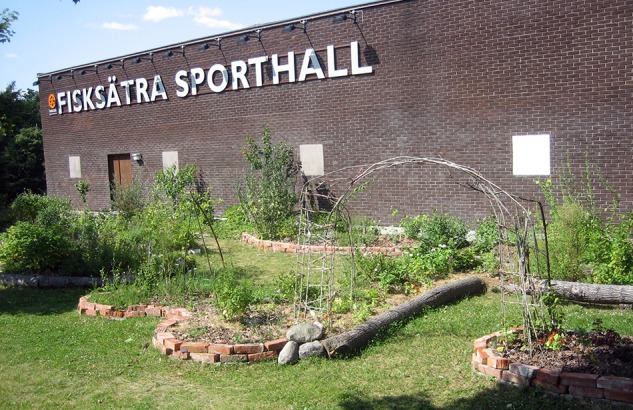
In front of the public sports hall in relatively low-income and culturally diverse suburb of Fisksätra, southeast of Stockholm. A lawn has been planted with around 130 different plant species to form an edible forest garden. On regular basis children from a nearby kindergarten visit the garden to learn about ecology and explore the affordances of the garden.

The high biodiversity could also strengthen the affordance for the Rich in species dimension and the perceived biodiversity, as already mentioned linked with use rates of urban green spaces (e.g., Sandifer et al., 2015). Again, when compared to traditional lawns, the potential difference here seems obvious (Figure 4). The emphasis on edibles in the forest garden does not exclude plants that are just there for aesthetic or other reasons (e.g., pest control or other functions), however, the edibility factor arguably offer even more ways to interact and relate to nature in meaningful ways using the whole body and all its senses. Forest garden environments may also support affordances important for species other than humans, such as singing birds, insects, and other animals that further may strengthen the Rich in species quality. Many times forest gardens also include the presence of an “insect hotel” – a structure made to provide shelter for insects – that in addition may contribute with, e.g., pollination functions.

Singing birds, sounds of wind blowing through the trees, etc. could also reinforce the affordances for the Serene dimension through the presence of various “sounds of nature” (Grahn and Stigsdotter, 2010). In addition, the potential to use trees and other vegetation to reduce, e.g., traffic noise levels have been highlighted in the literature (e.g., Bolund and Hunhammar, 1999; Gómez-Baggethun et al., 2013). Evidence also suggests that green features might mitigate annoyance associated with such noise in urban environments, and that the type and structure of the greenery matters in this regards. Li et al. (2010) for instance investigated Hong Kong residents and found “garden parks” visible from home to reduce noise annoyance to a greater degree than “grassy hills”. Renterghem and Botteldooren (2016) reached a similar result and concluded that visible outdoor vegetation was essential for the reducing effect on noise annoyance at home.

Trees and other perennials in the semi-open *multi-strata* structure (Figure 2) of the edible forest garden could furthermore support affordances for Refuge through the creation of shelters and hideaways. Such affordances have been described as particularly important from the perspective of stress restoration (Grahn et al., 2010; Pálsdóttir et al., 2017; Stigsdotter et al., 2017b). This has also been indicated in forest rehabilitation contexts (Sonntag-Öström et al., 2015). A sign and a gate that marks the entrance of the garden could further enhance such affordances (Pálsdóttir et al., 2017). If large enough to provide a sense of “coherent whole,” and of “entering a world in itself” (Grahn and Stigsdotter, 2010; Pálsdóttir et al., 2017), an edible forest garden may also afford of the perceived sensory dimension of Space to some extent.

Over time, an edible forest garden could also support opportunities to experience and appreciate the work and efforts of “previous generations,” thus affording the perceived sensory dimension of Culture (Grahn and Stigsdotter, 2010). Artifacts such as “sculptures” or “ornaments” (ibid), made with a sensibility for the qualities of the place could be used to further strengthen this dimension. A lawn would generally better afford the Prospect dimension than a forest garden in itself. However, from a distance a forest gardens could potentially aid in providing a pleasant “view” or “vista” that also are important aspects of this dimension (ibid). The potential of edible forest gardens to support various social affordances in urban areas, especially when implemented as community gardens, have already been pointed out above and will not be further discussed here. What may be important to highlight here though is the conflict that has been observed in empirical studies between highly restorative qualities such as Serene and the Social dimension (see e.g., Pálsdóttir et al., 2017). This implies that a balance is needed between the support of social affordances and the potential for restorative qualities in the forest garden if the environment is to support such opposing needs. For instance, this could be done by making sure that social activities in the garden is not overly promoted and that time slots are reserved where the garden can be available for those in need of a more solitary experience.

### How the Perceived Qualities of Edible Forest Gardens May Support Restorative Processes

Pálsdóttir et al. (2017) investigated the potential of a forest garden environment to support the rehabilitation process of individuals with stress-related mental disorders. Participants described how the “natural appearance” of the forest garden environment appealed to them and was perceived as “calming and safe.” Participants described how they felt that “nature was strongly present” in the forest garden, that “they could think without effort” and find a “way back to peace and quiet” (ibid). Other participants in this study mentioned the restfulness of the “overgrown and wild-like nature.” The forest garden environment was described as embedded in “lush vegetation” and participants mentioned how the wild attributes of the forest garden provided opportunities for “undemanding and restful” experiences. Some participants also mentioned regaining a feeling of “natural origin” and a strong “belonging to a greater whole” (ibid.). Participants in the study shared how they, in the forest garden environment, could “closely interact with nature” and “dared to expose their deepest feelings and thoughts.” The “smell of grass,” “the taste of berries,” the “sounds of the wind” and “bird twitter and songs” were other experiences mentioned. In the winter participants reported seeing tracks from animals in the snow, giving an indication that restorative processes may be supported during all seasons. The forest garden environment also allowed participants to “hide and find a nice, sheltered place” and “move around without being heard or seen.” Some participants “walked slowly or strolled around” in the forest garden, while others “just sat somewhere and enjoyed the surroundings” (ibid). Stigsdotter et al. (2017b) conclude that spatial aspects are important in order for environments to support restorative processes. Environments with a “natural and wild appearance,” “diverse vegetation,” and a “balance between enclosed, dense growth and open views” were found to be generally preferred in this regard. The dense growth should have “the appearance of a den and offer experiences of privacy” (ibid.). These are all descriptions that would suit the typical, mature forest garden well.

## Summary and Conclusion

We have highlighted several factors that present edible forest gardens based on agroecological principles as an interesting model to explore in order to achieve efficient multiple-use of urban green spaces. We have pointed to the potential of several ecological benefits from such a design and management strategy, not the least in terms of increased urban biodiversity, which could be achieved while simultaneously increasing affordances and perceived qualities important for human health and wellbeing. The global prevalence for diseases highly linked with lifestyle and living environment, in turn affected by increasing urbanization, stresses the importance of supporting such affordances in people’s close living environment. Not the least opportunities to restore from stress and attention fatigue seems important, but also possibilities to shape an increased sense of connectedness to nature and to processes of food production. This could also encourage pro-environmental behaviors that could further benefit long-term public health and wellbeing and mitigate ecological challenges. The importance of accessibility, not the least expressed in terms of physical proximity, for the perception and utilization of such green space affordances highlights the need to place edible forest gardens in public green spaces, at street level, close to dwellings and accessible for all. The general potential of green space affordances to mitigate socioeconomic differences in health and wellbeing can make edible forest gardens extra interesting to implement in socioeconomically challenged areas. Further research is encouraged in order to establish a deeper understanding for how affordances and qualities of salutogenic importance may be supported through urban green spaces and infrastructures. The potential of edible forest gardens in urban areas to contribute to biodiversity through the creation of new habitats, i.e., to also support affordances of importance for species other than humans may also be interesting to further investigate.

## Author Contributions

JS conceived to original idea for the paper and took responsibility for the overall structure and writing of the final manuscript. CS provided expertise and knowledge about urban sustainability, gardening, and edible forest gardens.

## Conflict of Interest Statement

The authors declare that the research was conducted in the absence of any commercial or financial relationships that could be construed as a potential conflict of interest.
